# A Mathematical Model for the Determination of Steady-State Cardiolipin Remodeling Mechanisms Using Lipidomic Data

**DOI:** 10.1371/journal.pone.0021170

**Published:** 2011-06-10

**Authors:** Lu Zhang, Robert J. A. Bell, Michael A. Kiebish, Thomas N. Seyfried, Xianlin Han, Richard W. Gross, Jeffrey H. Chuang

**Affiliations:** 1 Department of Biology, Boston College, Chestnut Hill, Massachusetts, United States of America; 2 Department of Biomedical Sciences, University of California San Francisco, San Francisco, California, United States of America; 3 Department of Internal Medicine, Washington University School of Medicine, St. Louis, Missouri, United States of America; 4 Diabetes and Obesity Research Center, Sanford-Burnham Medical Research Institute, Orlando, Florida, United States of America; Governmental Technical Research Centre of Finland, Finland

## Abstract

Technical advances in lipidomic analysis have generated tremendous amounts of quantitative lipid molecular species data, whose value has not been fully explored. We describe a novel computational method to infer mechanisms of *de novo* lipid synthesis and remodeling from lipidomic data. We focus on the mitochondrial-specific lipid cardiolipin (CL), a polyglycerol phospholipid with four acyl chains. The lengths and degree of unsaturation of these acyl chains vary across CL molecules, and regulation of these differences is important for mitochondrial energy metabolism. We developed a novel mathematical approach to determine mechanisms controlling the steady-state distribution of acyl chain combinations in CL . We analyzed mitochondrial lipids from 18 types of steady-state samples, each with at least 3 replicates, from mouse brain, heart, lung, liver, tumor cells, and tumors grown *in vitro*. Using a mathematical model for the CL remodeling mechanisms and a maximum likelihood approach to infer parameters, we found that for most samples the four chain positions have an independent and identical distribution, indicating they are remodeled by the same processes. Furthermore, for most brain samples and liver, the distribution of acyl chains is well-fit by a simple linear combination of the pools of acyl chains in phosphatidylcholine (PC), phosphatidylethanolamine (PE), and phosphatidylglycerol (PG). This suggests that headgroup chemistry is the key determinant of acyl donation into CL, with chain length/saturation less important. This canonical remodeling behavior appears damaged in some tumor samples, which display a consistent excess of CL molecules having particular masses. For heart and lung, the “proportional incorporation” assumption is not adequate to explain the CL distribution, suggesting additional acyl CoA-dependent remodeling that is chain-type specific. Our findings indicate that CL remodeling processes can be described by a small set of quantitative relationships, and that bioinformatic approaches can help determine these processes from high-throughput lipidomic data.

## Introduction

Phospholipids play a crucial role in biological systems. They act as key components in membrane physiology, bioenergetics, cellular recognition, and signal transduction [1]–[3]. While the phospholipid *de novo* biosynthesis pathway (the Kennedy pathway) is relatively well understood, there is still much to know about the remodeling pathway (the Lands cycle) [4]–[6]. The Lands cycle involves the deacylation/reacylation of existing cellular phospholipids to create new molecular species. Phospholipases, such as PLA_1_ and PLA_2_, remove existing acyl chains to generate lysophospholipids. Various transacylases or acyltransferases then reacylate the lysophospholipid with a donor acyl chain, changing phospholipid architecture, which provides a platform for numerous and diverse functional roles. Remodeling processes could in principle be complex, as there are an abundance of acyltransferase, transacylase, and phospholipase isoforms, which may be *sn*-1/*sn*-2 as well as acyl chain selective [6]. Characterization of lipid remodeling mechanisms is crucial to understanding the functional roles of lipids in biological systems.

Advances in mass spectrometry now allow for the high-throughput analysis of the cellular lipidome, which is comprised of numerous lipid classes as well as signaling intermediates [7]. The multidimensional mass spectrometry based shotgun lipidomics (MDMS-SL) approach is capable of simultaneously analyzing hundreds to thousands of lipid molecular species, providing tremendous amounts of data that can be used to infer mechanisms of lipid biosynthesis and remodeling in diseased and normal states [7]. However, bioinformatic tools and models to interpret MDMS-SL data are sparse [8], [9]. The further development of computational approaches to mechanistically analyze lipidomic data would be extremely valuable, in particular for analysis of cardiolipin (CL, 1,3-diphosphatidyl-sn-glycerol), a key polyglycerolphospholipid critically involved in energy metabolism, apoptosis, and membrane integrity [10].

CL is unique among phospholipids because it contains four fatty acid (FA) chains and is exclusively found in the inner mitochondrial membranes of eukaryotes [10], [11]. CL molecular species vary dramatically during development and among tissues. Abnormalities in these profiles have been associated with changes in cellular bioenergetics and diseases, including Barth syndrome, diabetes, heart failure, and cancer [11]–[16]. The distribution and arrangement of molecular species are thought to be achieved by acyl remodeling processes and have functional significance. Because the potential variety of CL species is extremely large (N^4^ positional permutations are possible with N types of fatty acids), CL distributions and identified molecular species have been difficult to analyze in numerous cases [15]. A systematic computational approach is in great need for the analysis and understanding of CL regulation.

CL is *de novo* synthesized from the condensation of phosphatidylglycerol (PG) and cytidine diphosphate-diacylglycerol (CDP-DAG). The four acyl chains of this immature CL are largely comprised of shorter and saturated or mono/diunsaturated acyl chains [12], [17], [18]. Immature CL species then undergo extensive remodeling. This can be achieved by an acyl CoA dependent deacylation-reacylation cycle, or via transacylation using acyl chains from the sn-2 position of phosphatidylcholine (PC) and phosphatidylethanolamine (PE) [10]. A limited number of enzymes involved in CL remodeling, such as CoA:lysocardiolipin acyltransferase (ALCAT1), monolysocardiolipin acyltransferase (MLCL AT), tafazzin, and calcium-independent phospholipase A2 (iPLA2) are known, though the specificities of these enzymes are not clear. There may also be many other remodeling enzymes yet to be discovered [18]–[23], and heterogeneity of lipids within the membrane may affect their activity [24].

Previously, we observed that the distribution of CL in the C57BL mouse (B6) brain could be qualitatively explained by a simple steady state model for CL remodeling [12], [25]. The model assumed random acyl chain incorporation into CL from a pool of PC (*sn*-2), PE (*sn*-2), and PG acyl chains, with each of the four CL chain positions assumed to be independently and identically remodeled [26]. Although the model was able to closely fit CL profiles for the mouse B6 brain, the model has not been formally described and it is not known whether the behavior is general for other tissues and diseased states.

In this work, we introduce a rigorous approach to systematically determine the CL remodeling mechanism in any sample at steady state. Using high throughput MDMS-SL data and a maximum-likelihood approach, we analyze 18 types of samples, from mouse brain, heart, lung, liver, tumor cells, and tumors grown in *vitro*. Improving on the assumptions of the simple model above, we built a two-step process to separately answer for any sample: whether the four chain positions of CL are independently and identically remodeled; and how the acyl donors (PC, PE, PG, and acyl CoA) contribute to CL fatty acid composition. Our method provides a fast and informative approach for testing hypotheses about CL remodeling mechanisms.

## Results

### Characterization of CL profiles across samples

**Figure 1:**
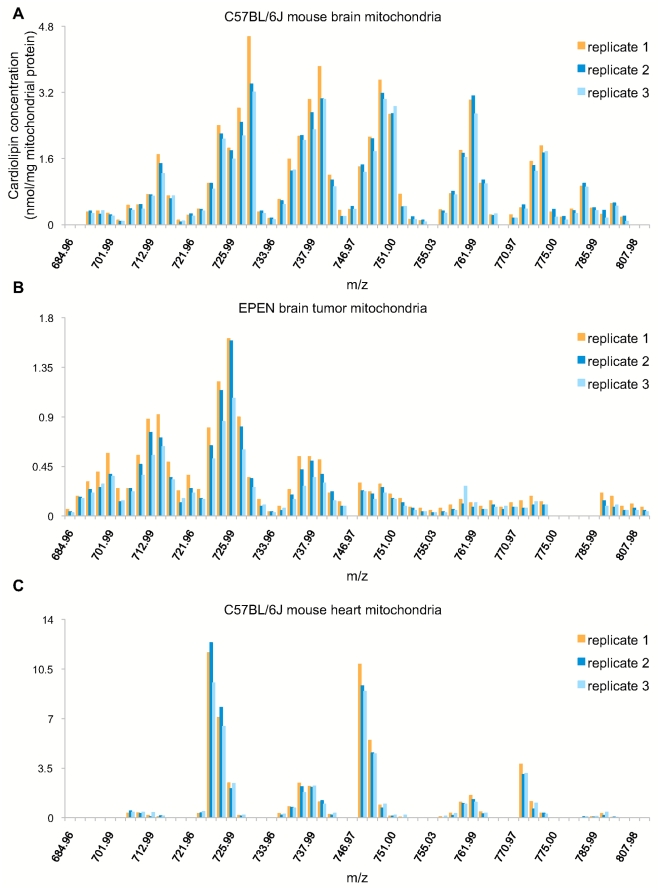
CL profiles are similar within biological replicates, but vary among samples/tissues. CL species are labeled along the x-axis by mass/charge ratio (m/z), and the y- axis shows MDMS-SL measured concentrations. C57BL/6J (B6) mouse brain mitochondria (A), EPEN brain tumor mitochondria (B), and B6 heart mitochondria (C) are shown as examples. The three replicates of each sample have similar CL distributions, but the three tissues have distinct CL profiles.

Highly purified mitochondrial lipidomic data were obtained from [12], [27]. 14 mouse brain samples were analyzed, including two normal strains C57BL/6J (B6) and VM/Dk (VM, which has a 210-fold increase in spontaneous brain tumor formation) [17]; B6 derived astro- cytoma tumor (CT-2A) and ependymoblastoma tumor (EPEN); VM derived stem cell tumor VM-NM1; and two microgliomas (VM-M2 and VM-M3). Tumors were grown *in vivo* and cell cultures *in vitro* [28], [29]. Two additional cell lines, astrocyte (non-tumorigenic) and BV2 (microglia), were used as controls. We also analyzed B6 mouse tissues from heart, lung, and liver. For each sample, the mass content and distribution of lipid molecular species (CL, PC, PE, PG) were quantified, with acyl CoA additionally measured for the B6 brain, heart, lung, and liver samples (All raw data are in Table S1). The sn-1 and sn-2 acyl chain designations of diacyl phospholipids (PC, PE, and PG) were determined by MDMS-SL analysis [30]. In general, CL profiles are consistent in replicates of a sample, but are diverse among different samples and tissues. Figure 1 shows a comparison of the samples B6 brain mitochondria, EPEN brain tumor mitochondria, and B6 heart mitochondria. The data suggest wide variations in lipid synthesis and remodeling processes across samples.

### Testing of the Independent and Identical Distribution Model

Our MDMS-SL procedure is capable of detecting more than 100 mass peaks of CL, each containing tens to hundreds of possible CL isomers. Given the potential complexity of processes affecting these CL species, we first analyzed the data from the perspective of CL fatty acid chain concentrations, an approach that reduces the dimensionality of the problem. As an initial question, we investigated whether the four acyl chain positions of CL are independently and identically remodeled. To test this, we built an “Independent and Identical Distribution” (IID) model, in which the relative frequency of a CL isomer is the product of the probabilities of its four fatty acid chains (Methods, Equation 1). The probability of a CL molecular species is the sum over all isomers with the same number of total carbons and double bonds (Methods, Equation 2). The FA distribution at the CL chain positions, {P_CL_(α_1_), P_CL_(α_2_), …, P_CL_(α_n_)} (α_i_ are chain types found among acyl donors PC sn-2, PE sn-2, PG, and acyl CoA), is predicted by minimizing the error between the predicted and observed CL molecular species distribution (Methods, Equation 3). If IID behavior is consistent with the remodeling processes within a sample, there should exist a FA distribution that can reproduce the experimentally observed CL profile when Equation 1 is applied.

Table 1 shows the least error and Pearson correlation coefficient between the observed and optimally fit CL distribution. 13/14 mouse brain samples yield r>0.7. A second set of B6 measurements for brain, heart, lung, and liver also yield r>0.9. The results suggest that in general, the four chain positions of CL are independently and identically remodeled. To test the robustness of the IID model, we performed four-fold cross validation. The correlation coefficients in the cross-validation were still strongly positive, though somewhat lower than for the original data. This is not unexpected since each CL peak contains information about only a few FA chains, and the cross-validation uses only subsets of the peaks at a time. However all samples and tissues achieved much higher correlation coefficients compared to random label-permuted data (t-test p-value<0.05) indicating that the IID model could accurately describe CL remodeling behavior. The observed and optimal predicted CL distributions are given for each sample in Table S2.

**Table 1:** The independent and identical distribution model successfully predicts CL distributions.

Sample	Error	Pearson Correlation	Pearson Correlationin cross validation (p-value)
BV2 *vitro*	0.0088	0.8465	0.49±0.17 (0.0032)
B6	0.0010	0.9845	0.96±0.01 (4.0E-05)
CT2A *vitro*	0.0053	0.9158	0.77±0.04 (1.4E-05)
Astrocyte *vitro*	0.0061	0.9206	0.46±0.27 (0.018)
EPEN *vitro*	0.0043	0.9205	0.75±0.03 (0.0018)
VM M2 *vitro*	0.0067	0.8451	0.65±0.05 (0.0075)
VM M3 *vivo*	0.0158	0.7416	0.36±0.09 (0.0012)
VM M3 *vitro*	0.0192	0.5804	0.31±0.09 (0.0068)
VM NM1 *vivo*	0.0126	0.8828	0.36±0.10 (0.0031)
VM NM1 *vitro*	0.0059	0.8908	0.73±0.12 (0.00037)
VM	0.0143	0.8437	0.66±0.09 (0.00019)
EPEN *vivo*	0.0080	0.8736	0.58±0.12 (0.00062)
VM M2 *vivo*	0.0150	0.7722	0.60±0.06 (0.00034)
CT2A *vivo*	0.0213	0.7245	0.39±0.20 (0.019)
B6 brain	0.0014	0.9745	0.91±0.01 (4.0E-06)
B6 heart	0.0007	0.9969	0.78±0.16 (0.00054)
B6 lung	0.0030	0.9813	0.93±0.01 (9.0E-06)
B6 liver	0.0002	0.9997	0.99±0.00 (3.6E-08)

Quality of fit was assessed by squared-error between the fit and observed CL distributions (column 2) as well as the Pearson correlation *r* of the fit and observed CL data (column 3). 17/18 samples show *r*>0.7. The 4th column shows Pearson correlation of the fit and observed CL distributions in four-fold cross-validation (mean ± standard deviation). P-values compare the cross-validation correlation values to correlations calculated on CL data with the labels randomly permuted (1-tail t-test). All samples achieved p-value<0.05 indicating that the IID model could correctly describe the CL remodeling system.

**Figure 2:**
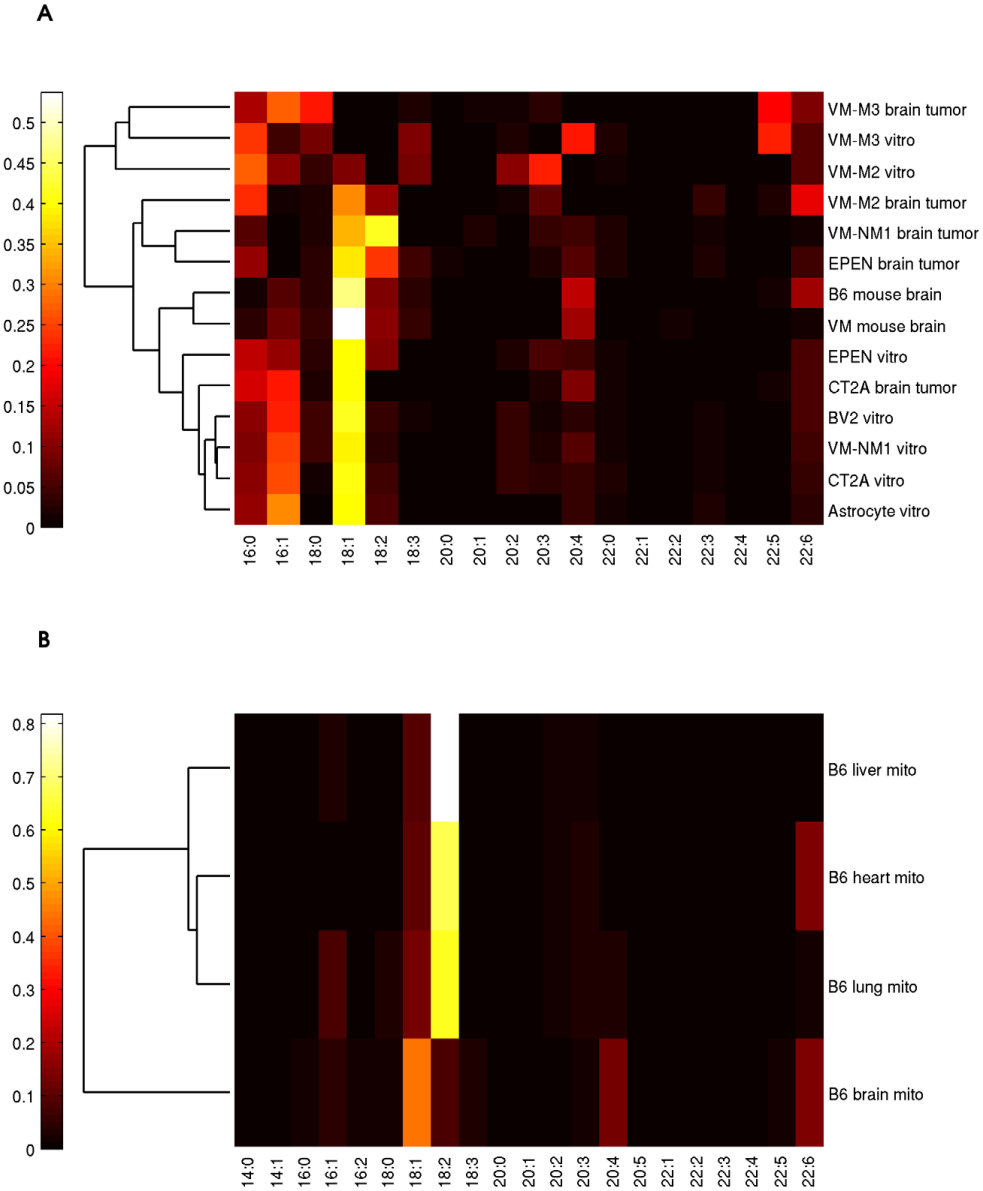
Comparison of CL FA compositions between 14 mouse brain samples (A) and 4 B6 mouse tissues (B). 18∶1 is predominant in brain (∼40%), while its percentage is reduced in some tumor samples (e.g. VM-M2 *vitro*, VM-M3 *vivo* VM-M3 *vitro*). 18∶2 is predominant in heart, lung, and liver (∼70%).

The predicted FA compositions of CL for each tissue are shown in Figure 2 organized into clusters. According to our inference the predominant FA in mouse brain is 18∶1 (∼48%). This is in contrast to heart, liver and lung, for which 18∶2 is the predominant component (∼70%). However, 18∶1 is reduced in tumor samples, in particular lower in VM-M2 *vitro*, VM-M3 *vivo*, and VM-M3 *vitro*. We also observed that the level of 16∶0 is elevated in tumor samples and cell cultures (>10%), compared to that in the B6 and VM brain samples (∼2%).

### Proportional Incorporation of Acyl Chains from Acyl Donors

To investigate the formation of acyl chain compositions in CL, we then tested a “proportional incorporation” model which is a refinment of the IID model. The proportional incorporation hypothesis is that that fatty acid incorporation rates are controlled by the head group of the donor class (PC, PE, PG or acyl CoA) and that FAs within an acyl donor class are indistinguishable to the remodeling enzymes. Under such a mechanism FAs would be incorporated into CL proportionally to their prevalence in the donor class. If this hypothesis is correct, the number of parameters in the CL remodeling system can be reduced to the number of acyl donor classes.

For the brain samples, we investigated a model with weight parameters W_PC_, W_PE_, and W_PG_ (Methods, Equation 6). These parameters indicate the relative contributions of each lipid class to CL FA composition in steady state. For each sample, we then searched for optimal parameter values that could explain the CL acyl chain composition. Inferred CL acyl compositions and experimental compositions in acyl donors are in Table S3. We successfully found weight parameters for 11/14 mouse brain samples with strong correlation coefficient (r>0.7) and significant p-values (p<0.05) (Table 2). We also investigated the proportional incorporation model with an additional acyl CoA weight parameter for the heart, lung, liver, and additional B6 brain sample, for which experimental acyl CoA distributions were available. Among these tissues, liver and brain showed a good fit (r>0.7) with the proportional incorporation model. Thus the simple proportional incorporation model describes CL remodeling in most tissues, despite the varying CL profiles in different samples/tissues. This suggests that PC and PE transacylation remodeling has little chain specificity within each class. In brain and liver, transacylation is the dominant remodeling process, as the inferred acyl CoA contribution is zero.

**Table 2:** Proportional incorporation model: inferred remodeling parameters and performance.

Sample	W_PG_	W_PC_	W_PE_	W_AC_	Pearson	p-value (Pearson)	Error	p-value (Error)
BV2 *vitro*	0.4471	0.3021	0.2508	-	0.8608	0.0132	0.052	0.0154
B6	0	1	0	-	0.8936	0.0024	0.0494	0.0024
CT2A *vitro*	0	1	0	-	0.9117	0.0041	0.0361	0.0038
Astrocyte *vitro*	0.0956	0.9044	0	-	0.8868	0.0064	0.0538	0.0075
EPEN *vitro*	0.2767	0.7128	0.0105	-	0.9072	0.0012	0.0304	0.0008
VM M2 *vitro*	0.3741	0.3641	0.2619	-	0.4711	0.1468	0.1089	0.2433
VM M3 *vivo*	0	0.7419	0.2581	-	−0.1189	0.8255	0.2201	0.9722
VM M3 *vitro*	0	0.4694	0.5306	-	0.1829	0.4544	0.1572	0.6088
VM NM1 *vivo*	0.2284	0.7716	0	-	0.9428	0.0002	0.0451	0.0016
VM NM1 *vitro*	0.06	0.94	0	-	0.8619	0.0143	0.0464	0.0091
VM	0.4823	0.481	0.0367	-	0.7726	0.0143	0.1105	0.0111
EPEN *vivo*	0.4261	0.3887	0.1852	-	0.9384	0	0.0258	0
VM M2 *vivo*	0.5701	0	0.4299	-	0.9249	0.0004	0.0211	0.0002
CT2A *vivo*	0.6631	0.0262	0.3108	-	0.8498	0.0038	0.0532	0.0029
B6 brain	0.2869	0.7131	0	0	0.8893	0.0042	0.0415	0.0039
B6 heart	0	0.2212	0	0.7788	0.5069	0.2267	0.3358	0.2313
B6 lung	0	1	0	0	0.5490	0.1724	0.2624	0.1647
B6 liver	0	1	0	0	0.7859	0.0157	0.2889	0.0131

The optimal remodeling weights inferred via the proportional incorporation model are given for each sample for PC, PE, and PG, as well as for acyl CoA for the four tissues where acyl coA data were available. The quality of fit was assessed by Pearson correlation *r* and squared-error between the fit and observed CL acyl compositions (columns 6 and 8). Most samples were successfully fit by the proportional incorporation model (r>0.7). P-values were assessed by comparing to results for label-permuted PC, PE, PG, and acyl CoA FA data (columns 7 and 9).

In 10/18 samples, PC had the largest contribution to the CL acyl chain compositions. PE had the largest contribution in only 1/18 samples, and it had the lowest contribution in 13/18. For the four B6 tissues, our inference suggests that acyl CoA is most relevant in heart.

### Deviations from the Proportional Incorporation Model

**Figure 3:**
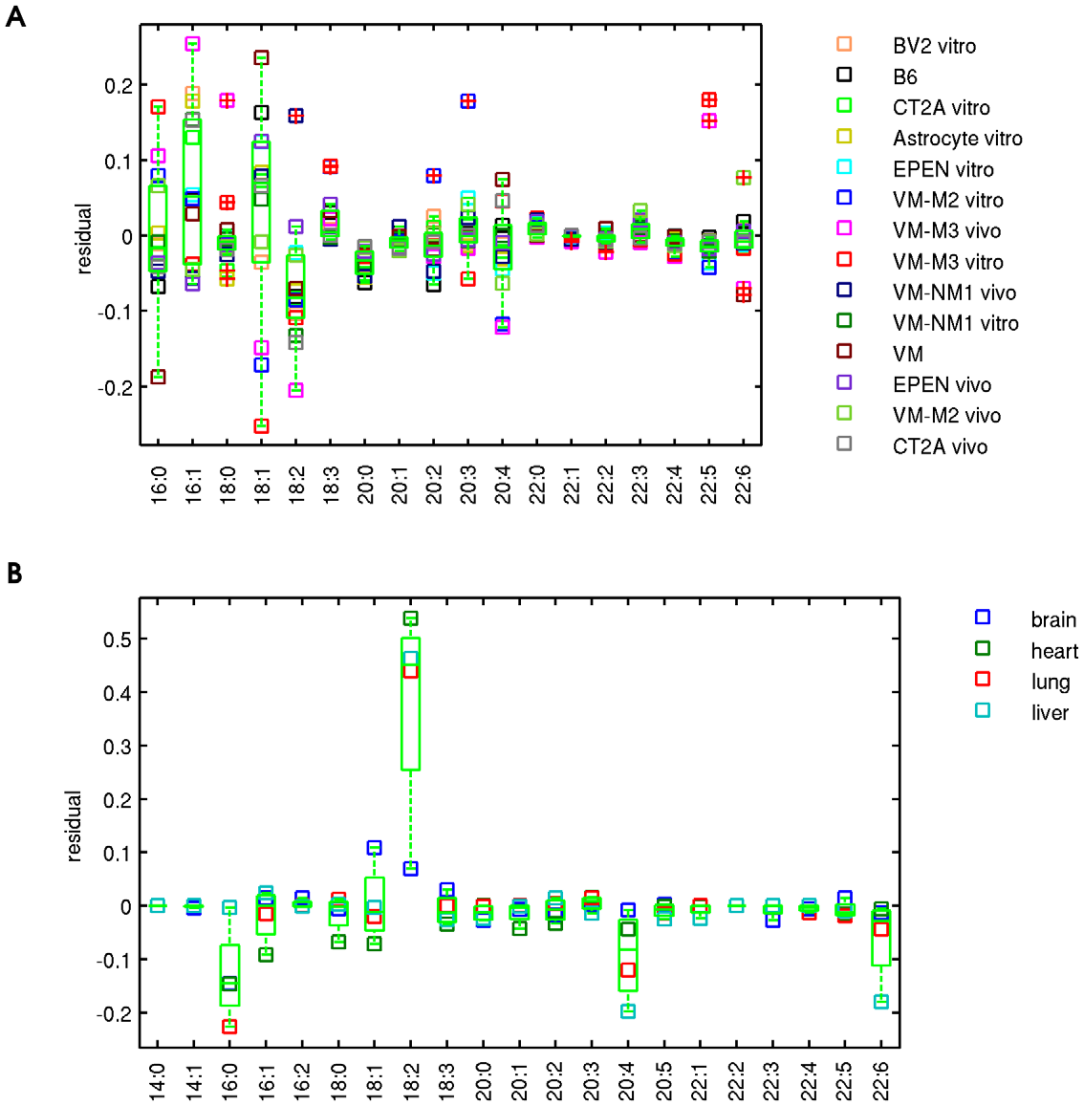
FA residuals reveal potential enzyme selectivity. The y-axis shows the residual between the FA composition best describing the CL data and the fit to this FA composition by the proportional incorporation model. A positive residual suggests enzyme preference. Box statistics are shown for 14 brain samples (A) and 4 B6 mouse tissues (B) with median and 25^th^ and 75^th^ percentiles.

For samples where the proportional incorporation model failed to explain the CL profile (VM-M3 vivo r = −0.1189, VM-M3 vitro r = 0.1829, VM-M2 vitro r = 0.4711, B6 heart r = 0.5069, B6 lung r = 0.5490), a number of explanations are possible. One possibility is that an additional remodeling enzyme has been utilized, altering the selectivity on chain types within a donor class. In order to gain insight into which individual chain types are under selection, we plotted FA composition residuals in the fit to the proportional incorporation model (Figure 3). A positive residual indicates that there is an excess of the chain in CL over that predicted by the proportional incorporation model, i.e. a remodeling enzyme may favor that chain type, and vice versa for negative residuals. In the VM-M3 vivo and VM-M3 vitro samples, 16∶0, 18∶0 and 22∶5 are favored in CL and 18∶1 is disfavored. 18∶2 is highly favored in B6 heart, lung, and liver, but not in brain. 16∶0, 20∶4 and 22∶6 are slightly disfavored across the four tissues. This residuals approach may be useful for understanding the behavior of remodeling enzymes in genetic perturbations.

**Figure 4:**
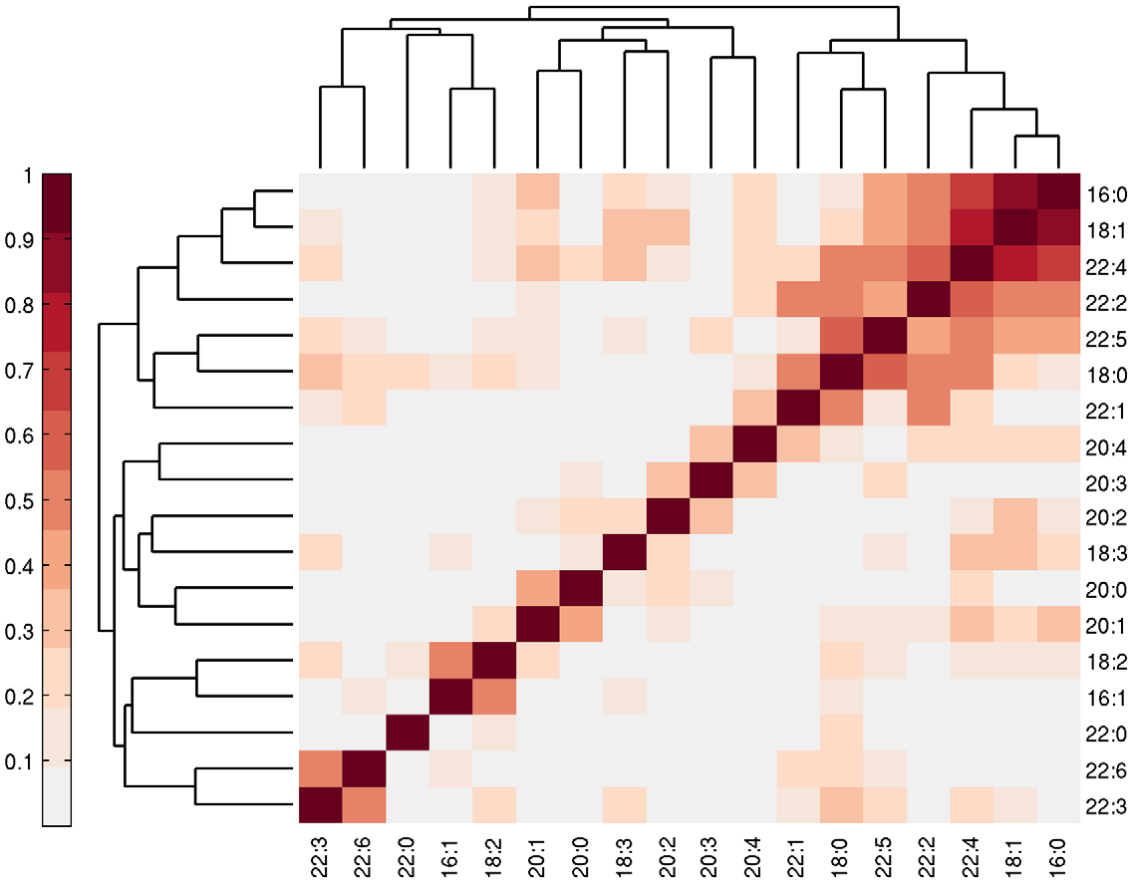
Hierarchical clustering of FAs suggests co-regulation. For each pair of FAs, we calculated the squared correlation of their residuals (data vs. proportional incorporation model) in the 14 brain samples. FAs were then hierarchically clustered. The chains {16∶0, 18∶1, 22∶4} show stronger clustering, suggesting they may respond to the same remodeling enzyme.

We also calculated the pair-wise correlation (R^2^) of residuals, and hierarchically clustered chain types. As shown in Figure 4, we found a group of chains {16∶0, 18∶1, 22∶4} that cluster with one another. This suggests that these chain types respond to the same selective remodeling enzyme.

## Discussion

We have presented a novel, powerful computational method to infer CL remodeling mechanisms from MDMS-SL data, which we expect to be critical for understanding CL metabolism. New mechanistic inference methods are of great importance since lipidomic data, such as those in the LIPID MAPS projects [31]–[33], are becoming increasingly abundant [34], [35]. We focused on synthesis and remodeling primarily within one class of lipids, CL, taking advantage of the accuracy of MDMS-SL identification of individual CL molecular species. In contrast, prior lipid computational analyses have tended to focus on broader characterizations of lipid pathways, such as the relative concentrations of lipid classes [36] or signal transduction leading to lipid changes [37]. Our focus on CL allowed us to investigate a mechanistic model having a very small number of fit parameters, which enabled us to robustly determine and cross-validate the remodeling behavior in each sample.

Although CL displays tissue- and sample-specific distributions, 17/18 samples can be explained by the IID model. The proportional incorporation model also fits 11/14 brain samples as well as the extra B6 brain and liver samples, which is surprising given the model's simplicity. The success of the proportional incorporation model suggests that in these tissues, the headgroups on acyl donors are the main determinants of contribution to CL, rather than selectivity on individual acyl chains. This is consistent with previous studies indicating that the activities of remodeling enzymes such as PLA2 are determined by interactions on the bilayer surface rather than the hydrophobic interior [38]. We also observe that PC in general contributes more to CL than the other donor classes. Although the mechanism for this is unclear, the classical surface dilution model [24] suggests this may be an indication of domains within the mitochondrial membrane of greater PC concentration, to which remodeling enzymes may bind more easily. If there is selectivity on the acyl chains, we speculate this may more commonly arise within acyl CoA-related mechanisms. This is because the proportional incorporation model works well even for many tissues for which acyl CoA data were not available, This is consistent with PC and PE contributing proportionally while acyl CoA is subject to more specific regulation.

Interestingly, when we did observe deviation from the IID behavior, these deviations had certain regularities. For example, the CL peaks at 80∶14 and 80∶15 are consistently underestimated in the BV2 *vitro*, VM M3 *vitro*, VM, and CT2A *vivo* samples (Figure S1). The regularity of such deviations suggests there are remodeling mechanisms that can distinguish the four positions or which involve positional dependencies.

One simple way the chain positions could be distinguished is by their *sn*-1 or *sn*-2 chemistry. We examined this by modifying the IID model to an “independent and differential distribution” (IDD) model that distinguishes *sn*-1 vs. *sn*-2 positions. However, this modification provided little improvement in the fit. Only the VM-NM1 *vivo* sample showed significant improvement (p-value<0.05; see Methods). This suggests that the unusual peaks at 80∶14 and 80∶15 are controlled by dependencies between positions, rather than independent but differential behavior. This unimportance of *sn*-1/*sn*-2 chemistry is surprising given that remodeling reactions acting on diacyl phospholipids (PC, PE, PG, PI) have a bias for the *sn*-2 position [39].

A minor caveat to our CL analysis is that it is based on the concentrations of CL peaks as defined by the number of carbons and double bonds among the acyl chains. Some lipidomic measurement methods such as MDMS-SL provide additional information about species distributions within each peak. Such information is irregular and with different uncertainties across peaks, making it difficult to use, but future methods may benefit from it.

Also, the model we have described here pertains to steady-state behavior. This is appropriate since all of the samples we have analyzed are under steady-state conditions. Therefore enzyme specificities impact our model as they would affect equilibrium constants in typical chemical systems (see Methods). Parameter inference for the dynamics of remodeling is a valuable future goal, and we have previously described software for simulating dynamic cardiolipin remodeling [27]. However, optimal solution of the dynamic problem is beyond the scope of this paper, as it requires experimental timecourse data and is also a more challenging statistical inference problem.

Because of the tradeoff between complexity and robustness of the inferred model [40], we have studied simplified models which are approximations of the behavior of CL. Aside from a generalization to handle dynamic data, further refinements may include parameters for more nuanced distinctions among donor classes. Such distinctions may be important for understanding individual enzymes such as *tafazzin*, which influences the transfer of chains from PC to CL [23]. Other important factors may include the relative positions of PC, PE, and PG in the CL remodeling pathway [10], [41], [42], molecular symmetry preferences [43], [44], or transfer of lipids among membranes. Through careful consideration of such refinements, we expect that our mechanistic inference method will generalize to other classes of lipids, providing a broad approach to analyzing diseases associated with alterations of lipid metabolism.

## Methods

### Sample Datasets

To compare CL remodeling between normal and pathological states, we analyzed 14 mouse brain samples, described in [12]. The samples were C57BL/6J (B6) and VM/Dk (VM) inbred mice; an astrocytoma (CT-2A) and an ependymoblastoma (EPEN) that are chemically induced from syngeneic B6 brain; two microgliomas (VM-M2 and VM-M3) and a stem cell tumor (VM-NM1) that arose spontaneously from the syngeneic VM brain; and two brain cell lines: astrocyte (non-tumorigenic) and BV2 (microglia). For each of these brain and brain tumor samples, three biological replicates were performed. To compare CL remodeling across tissues, we studied brain, heart, lung, and liver from C57BL/6J(B6) wild-type male mice (4 months of age), each with four biological replicates [27].

### MDMS-SL quantification of mitochondrial lipids

Extraction and quantification of the mitochondrial lipidome for these samples were previously described in [12], [27]. Briefly, tissue and tumor samples were harvested from mature male mice or cell cultures, and mitochondria were isolated and purified. An aliquot of purified mitochondria was transferred to a disposable culture borosilicate glass tube. Internal standards were added based on the protein concentration (in nmol/mg mitochondrial protein). Lipids from each mitochondrial homogenate were extracted by a modified Bligh and Dyer procedure. Each lipid extract was reconstituted with mitochondrial protein in chloroform/methanol. The lipids extracted were flushed with nitrogen, capped, and stored at −20°C. Each lipid solution was diluted prior to infusion and lipid analysis. Lipid molecular species were identified and quantified by high resolution MDMS-SL, which is described in detail in [30]. MDMS-SL allows for sn-1/sn-2 acyl chain determinations for diacyl species because sn-1 and sn-2 carboxylate groups fragment at differing rates in the mass spectrometry procedure. CL, PC, PE, PG, and acyl CoA (where available) concentrations were normalized to probability distributions and averaged among replicates.

### Independent and identical distribution model

All of our models assume that data reflect steady state behavior [27]. Although remodeling is dynamic, we are able to model steady-state behavior in the same way that chemical equilibria can be modeled without knowledge of individual reaction rates.

The IID model posits that four CL chain positions (*sn*-1, *sn*-1′, *sn*-2, *sn*-2′) are independently and identically remodeled. Therefore each position is assumed to have the same FA distribution. The concentrations of each FA chain are the parameters to be inferred for each sample. We implemented a maximum-likelihood approach to infer these parameters. Let 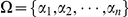 be all of the possible chain types in CL. The possible chain types are assumed to be those observed within the acyl donors PC, PE, and PG (and acyl CoA when available) in the sample.

Given the FA composition parameters in CL 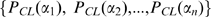, the IID probability of the CL isomer with acyl chain α_i_ at position *sn*-1, α_j_ at position *sn*-1′, α_k_ at position *sn*-2, and α_h_ at position *sn*-2′ is defined by Equation 1:
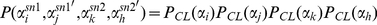
(1)


MDMS-SL measures the total concentration of CL molecular species with c carbons and d double bonds in the acyl chains. The corresponding IID probability is summed from the isomers matching c and d, as given by Equation 2: 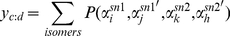
(2)


The FA distribution can be found by a maximum likelihood approach which compares 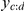 with the experimentally derived relative concentrations 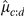. The likelihood of observing the data (for all CL species, S = 1..N) given the model and parameters is assessed by the error between the predicted and observed CL distributions, Equation 3:
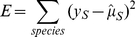
(3)


Minimizing the error function is equivalent to maximizing a likelihood function in which deviations from the experimental value are distributed according to a Gaussian probability function. We assume the variance associated with each Gaussian to be identical, which is reasonable since this is determined by the measurement uncertainty of the general MDMS-SL procedure for CL. The optimal FA compositions are those that yield the minimum error between the predicted and observed CL distributions, as described in Equation 4:
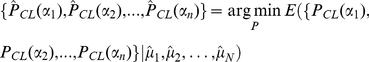
(4)


We searched for optimal parameter values iteratively using the Matlab function *lsqcurvefit*, with an initial condition of uniform probabilities (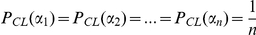), and under the constraints that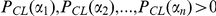 and 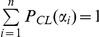.

Least error and Pearson correlation between the optimal predicted and observed CL probability distributions were used to evaluate the performance of the model. These gave similar results. We set a threshold correlation coefficient of 0.7 to decide whether the model explained the sample data.

### Four-fold cross-validation

The measurable CL molecular species were randomly divided into four disjoint subsets of equal size. Three subsets were used to train the model and the last was used for testing. The training-predicting procedure was repeated four times, each time leaving out a different subset. We then merged the predictions for the four test subsets and compared the resulting predicted CL distribution to the observed data using Pearson correlation coefficient. For the calculations of Table 1, the correlation was calculated in three separate runs, where in each run the subsets of the CL data were divided by a different randomization.

### Label permutation test

We performed a permutation test to assess the significance of the IID model's performance. For each sample, we generated a randomized version of the data by permuting the labels on CL concentrations. Our null hypothesis was that there is no dependency between the features (FA compositions) and the labeled values (CL species concentrations). We performed four-fold cross validation on the randomized dataset and repeated this procedure three times using different randomized labeling. A t-test was used to assess significance of the correlation coefficient for the original data.

### Independent and differential distribution model

This model tests whether *sn*-1/*sn*-1′ and *sn*-2/*sn*-2′ positions of CL are differentially remodeled, maintaining the assumption that the four positions of CL are independent. It is similar to the IID model, except having distinct FA compositions: 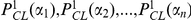 for *sn*-1/*sn*-1′ positions and 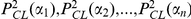 for *sn*-2/*sn*-2′ positions. The probability of the CL isomer with α_i_ at position *sn*-1, α_j_ at position *sn*-1′, α_k_ at position *sn*-2, and α_h_ at position *sn*-2′ is defined by Equation 5:
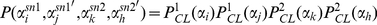
(5)


The parameter set 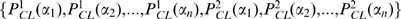 is optimized in the same manner as for the IID model. To compare the IDD and IID, we consider the results of the four-fold cross-validation for each model. For a given sample, we perform the four-fold cross validation for both IDD and IID in three runs each. Cross-validation requires a random data splitting, so for each run we split the data differently using a random number generator. The observed correlation in the cross-validation is an indication of the robustness of the model. To quantify IDD vs. IID we compare the distribution of correlation scores (see Methods: four-fold cross-validation) for the three runs of the two models, using a t-test.

### Proportional incorporation model

The model assumes FAs from an acyl donor class are incorporated into CL at rates proportional to their prevalence within the donor class. This is equivalent to assuming that there are no chain-type specificities of remodeling enzymes, i.e. transacylases that transfer acyl chains from PC and PE would have specificity for only the PC/PE head group and not the length or saturation of the acyl chain. In this model, the relative concentration of acyl chain 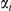 in CL is a weighted sum of the relative concentrations of the chain in each donor class (Equation 6). For PC and PE, we allow only their *sn*-2 acyl chains to contribute to the acyl chain pool, consistent with prior literature [45]–[47]. The weight parameters 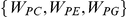
indicate their relative contributions to CL, representing the overall effect of phospholipid concentration and transacylase/phospholipase activities [48]. For example, if the affinity weights are W_PC_ = 0.5, W_PE_ = 0.25, and W_PG_ = 0.25, this means that of all the acyl chains making up the CL species distribution, 50% originate from PC (*sn*-2) and 25% are derived from each of PE (*sn*-2) and PG. 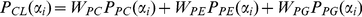
(6)


The optimal parameter values can be found by constrained linear regression. The weight values are related to the observed CL concentrations via:
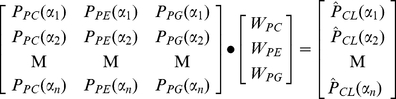
(7)Here the relative concentrations of acyl chains in each of PC sn-2, PE sn-2, and PG (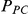,
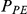
_ and_ 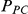) are experimentally determined. The acyl distribution within CL (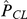) is predicted using the IID model, since experimental measurements for it were not readily available. We use Matlab function *lsqlin* to infer optimal parameter values, which minimizes the error 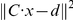, with constraints 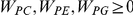 and 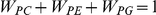. The residual for a given chain is defined to be that chain's component of 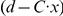.

For B6 brain, heart, lung, and liver analysis, we additionally include acyl CoA as an acyl donor, with weight parameter 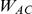 (Equation 8):
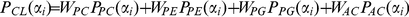
(8)


To assess the significance of model behavior, for each sample, we randomly permuted PC, PE, and PG FA compositions (each column of 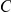), and generated 10000 different random datasets. An empirical p-value was assigned for the correlation coefficient of the true data based on the distribution of coefficients in the random datasets.

Note that all of the models we have described are consistent with a dynamical system at steady state. To clarify this relationship, we describe the corresponding dynamic system for the Proportional Incorporation model, The IID and IDD models are analogous. The dynamic model is similar to one we previously described in [27]. Under a model in which all four acyl chain positions behave equivalently, the dynamics of acyl chain type 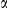 in CL are given by the equation:
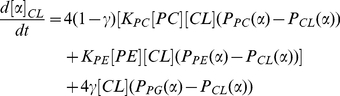
(9)



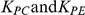
 are rate constants for the transfer of acyl chains from PC or PE sn-2 chains into CL, respectively. 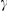 is the degradation rate constant for CL. 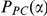 is the fraction of PC chains that can be transferred into CL that are of type α (with similar definitions for 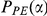 and 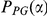). 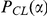 is the fraction of all CL chains that are of type α. Since we have assumed that the total concentration of CL (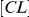) is in steady state, this obviates the need for an explicit synthesis rate constant 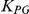.

The probability of chain type 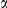 in Cardiolipin is:
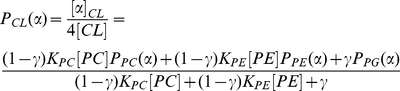
(10)This is equivalent to Equation 6, where
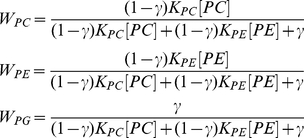
(11)


To further clarify the methods, we have provided a supplementary file providing a walk-through of the methods as applied to toy data (Methods S1).

## Supporting Information

Figure S1 **Trends of deviation from the IID model.** Certain CL species appear to consistently deviate from the IID model, notably 80∶14 and 80∶15 (marked with *). (A) BV2 *vitro* (r = 0.8465), (B) VM M3 *vitro* (r = 0.5804), (C) VM (r = 0.8437) and (D) CT2A *vivo* (r = 0.7245).
(TIF)

Table S1 **PC, PE, PG, acyl CoA, and CL concentrations as measured by MDMS-SL.**
(XLS)

Table S2 **Observed and model-predicted cardiolipin distribution for 14 samples and B6 tissues.**
(XLS)

Table S3 **Inferred CL acyl compositions and experimental compositions in acyl donors PG, PC** ***sn*** **-2, PE** ***sn*** **-2, and acyl CoA.**
(XLS)

Methods S1 **Example illustrating the mathematical methods described in the paper.**
(DOC)
