# Supplementary figures and images for: A Mathematical Model for the Determination of Steady-State Cardiolipin Remodeling Mechanisms Using Lipidomic Data
Source: PLoS One. 2011 Jun 10;6(6):e21170. doi: 10.1371/journal.pone.0021170 (PMC3112230; doi:10.1371/journal.pone.0021170)

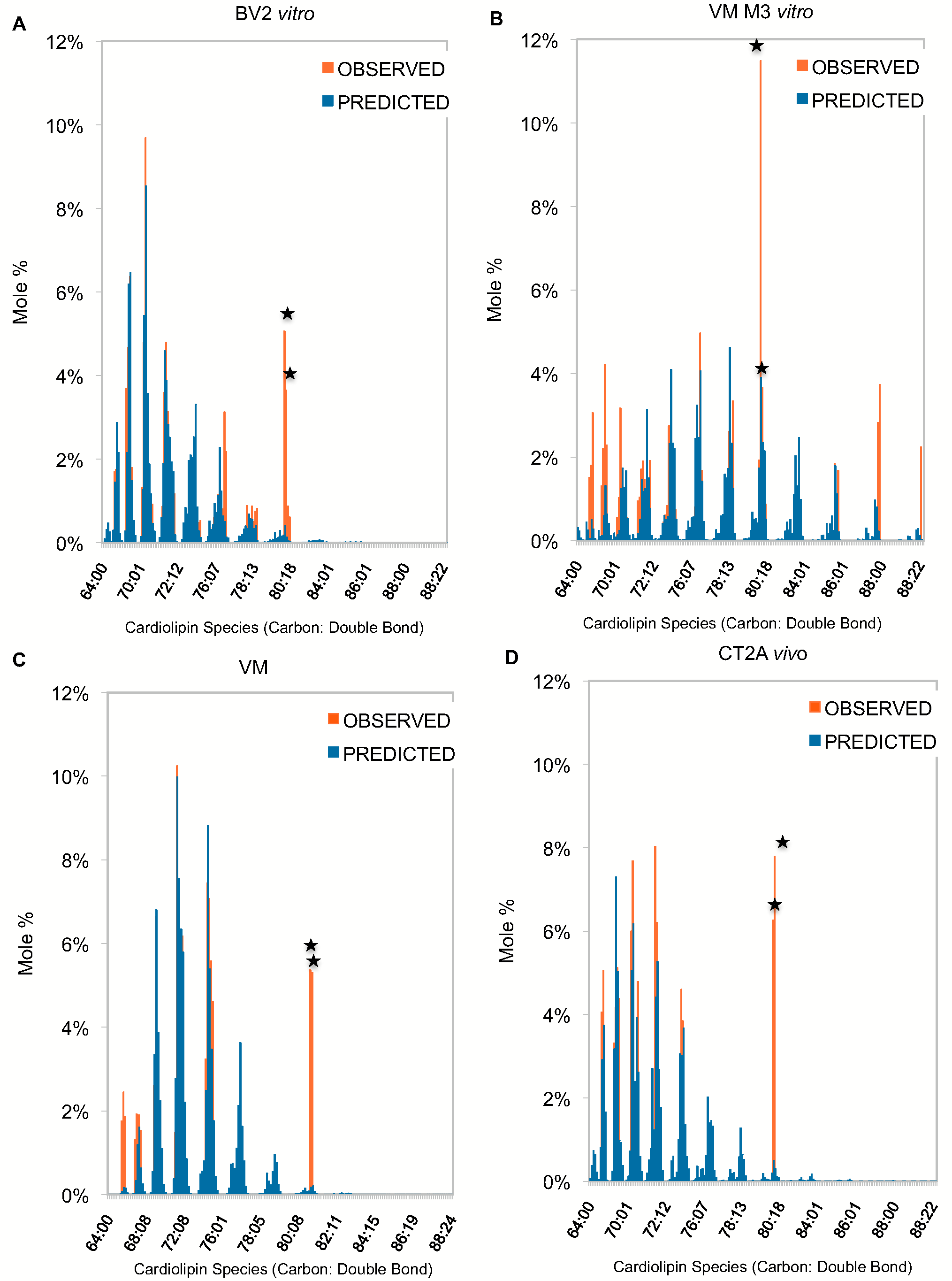

Supplement: Figure S1 — Trends of deviation from the IID model. Certain CL species appear to consistently deviate from the IID model, notably 80∶14 and 80∶15 (marked with *). (A) BV2 vitro (r = 0.8465), (B) VM M3 vitro (r = 0.5804), (C) VM (r = 0.8437) and (D) CT2A vivo (r = 0.7245). (TIF) [file pone.0021170.s001.tif]
