# Supplementary material for: A Mathematical Model for the Determination of Steady-State Cardiolipin Remodeling Mechanisms Using Lipidomic Data
Source: PLoS One. 2011 Jun 10;6(6):e21170. doi: 10.1371/journal.pone.0021170 (PMC3112230; doi:10.1371/journal.pone.0021170)
Supplement: Methods S1 — Example illustrating the mathematical methods described in the paper. (DOC) [file pone.0021170.s005.doc]

**Supplementary method**

**An example**

Let $\Omega$ be all possible fatty acid chain types in CL. Let $S$ be all possible CL molecular species given $\Omega$. In this example, we set and.

Assume we measured the following CL molecular species concentrations using MDMS-SL:

| CL molecular species  (carbon: double bond) | All possible isomers  (sn-1, sn-1’, sn-2, sn-2’) | Replicate 1  Concentration (nmol/mg protein) | Replicate 2  Concentration (nmol/mg protein) |
| --- | --- | --- | --- |
| 64:0 | 16:0-16:0-16:0-16:0 | 0.2 | 0.1 |
| 66:2 | 18:2-16:0-16:0-16:0  16:0-18:2-16:0-16:0  16:0-16:0-18:2-16:0  16:0-16:0-16:0-18:2 | 1.3 | 1.4 |
| 68:4 | 16:0-16:0-18:2-18:2  16:0-18:2-16:0-18:2  16:0-18:2-18:2-16:0  18:2-16:0-16:0-18:2  18:2-16:0-18:2-16:0  18:2-18:2-16:0-16:0 | 5.0 | 5.4 |
| 70:6 | 16:0-18:2-18:2-18:2  18:2-16:0-18:2-18:2  18:2-18:2-16:0-18:2  18:2-18:2-18:2-16:0 | 11.5 | 11.7 |
| 72:8 | 18:2-18:2-18:2-18:2 | 8.1 | 8.8 |
| Total |  | 26.1 | 27.4 |

Compute relative concentrations of CL molecular species and average among replicates to obtain the observed probability distribution.

For example,

| CL molecular species | Observed probability distribution |
| --- | --- |
| 64:0 | 0.006 |
| 66:2 | 0.050 |
| 68:4 | 0.194 |
| 70:6 | 0.434 |
| 72:8 | 0.316 |

**Independent and identical chain model**

To test the “independent and identical” hypothesis, let FA compositions of CL be $P_{\text{CL}}(18:2)=p$and $P_{\text{CL}}(16:0)=1-p$.

According to Equation (1) and (2), the IID probabilities of the CL molecular species are:

The optimal parameter value can be computed by minimizing the error between the predicted$(y_{c:d})$ and observed $(\hat{m}_{c:d})$ probability distributions of CL molecular species, Equation (3). Using matlab function *lsqcurvefit*, we obtain and . The optimal predictions are the following:

| CL molecular species | Predicted probability distribution | Observed probability distribution |
| --- | --- | --- |
| 64:0 | 0.0037 | 0.006 |
| 66:2 | 0.0450 | 0.050 |
| 68:4 | 0.2068 | 0.194 |
| 70:6 | 0.4218 | 0.434 |
| 72:8 | 0.3227 | 0.316 |

To evaluate the goodness of fit, Pearson correlation coefficient is calculated between the predicted and observed distributions. We get $r=0.9985$, suggesting the model could probably describe the system correctly.

More rigorously, cross-validation is performed. Here we use leave-one-out cross-validation as a demonstration. Parameter value is optimized from four training species and used to predict the probability of the test species. Pearson correlation coefficient is calculated between the predicted and observed probability distributions as above. We get .

| Training set | Test species | Predicted distribution | Observed distribution |
| --- | --- | --- | --- |
| {66:2,68:4,70:6,72:8} | 60:4 | 0.0037 | 0.006 |
| {60:4,68:4,70:6,72:8} | 66:2 | 0.0448 | 0.050 |
| {60:4,66:2,70:6,72:8} | 68:4 | 0.2118 | 0.194 |
| {60:4,66:2,68:4,72:8} | 70:6 | 0.4218 | 0.434 |
| {60:4,66:2,68:4,70:6} | 72:8 | 0.3351 | 0.316 |

Four-fold cross-validation was used in real data analysis. Multiple runs were performed using different random divisions of four fold subsets.

**Permutation Test**

The observed probabilities of CL molecular species are shuffled, resulting in multiple versions of random data.

| CL molecular species | Random data 1 | Random data 2 | Random data 3 |
| --- | --- | --- | --- |
| 64:0 | 0.434 | 0.194 | 0.050 |
| 66:2 | 0.194 | 0.006 | 0.194 |
| 68:4 | 0.316 | 0.434 | 0.316 |
| 70:6 | 0.006 | 0.316 | 0.006 |
| 72:8 | 0.050 | 0.050 | 0.434 |

Leave-one-out cross-validation is implemented on each random dataset. Pearson correlation coefficient values are calculated and shown in the following table:

|  | Random data 1 | Random data 2 | Random data 3 |
| --- | --- | --- | --- |
|  | 0.2937 | 0.5218 | -0.3246 |

The performances on random data are generally worse than on the original data. A one-tail z-test is performed comparing $r_{\text{original}}$ and $r_{\text{random}}$. P-value = 0.0285, which is below the significant threshold 0.05, suggesting the model performance is not a random behavior. In real data analysis, multiple runs of four-fold cross-validation results were considered thus t-test was used instead of z-test. More random datasets are preferable for a valid statistical test.

**Proportional Incorporation model**

Molecular species concentrations and sn-1/sn-2 acyl chain designations are measured by MDMS-SL for PC, PE, and PG. The FA compositions of PC sn-2, PE sn-2, and PG are computed individually. For example, given the following PC concentrations:

| PC molecular species  (sn-1, sn-2) | Concentration  (nmol/mg protein) |
| --- | --- |
| 16:0-16:0 | 17 |
| 16:0-18:1 | 10 |
| 18:1-18:1 | 24 |
| 18:1-18:2 | 4 |
| 16:0-20:4 | 19 |
| 18:2-22:6 | 26 |
| Total | 100 |

…

The probabilities of FA in CL can be computed using the IID model since they are not readily available. Then we have the following lipid FA distribution table:

| Acyl chain type | CL | PC sn-2 | PE sn-2 | PG |
| --- | --- | --- | --- | --- |
| 16:0 | 0.20 | 0.17 | 0.07 | 0.32 |
| 18:1 | 0.48 | 0.34 | 0.13 | 0.51 |
| 18:2 | 0.04 | 0.04 | 0.01 | 0.01 |
| 20:4 | 0.09 | 0.19 | 0.27 | 0.07 |
| 22:6 | 0.19 | 0.26 | 0.52 | 0.09 |

To test the proportional incorporation hypothesis, we use a set of weight parameters , subject to the constraints that and , indicating the relative contributions from each acyl donor to the CL FA compositions, Equation (6). Parameter values are optimized using linear regression:

Using matlab function *lsqlin*, we obtain the optimal weights . The Pearson correlation coefficient between predicted and observed CL FA distributions is $r_{\text{original}}=0.97$.

**Permutation test**

To evaluate the robustness of the model, FA compositions of each acyl donor lipid are shuffled, resulting in random datasets. For example:

| Acyl chain type | PC sn-2 | PE sn-2 | PG |
| --- | --- | --- | --- |
| 16:0 | 0.26 | 0.13 | 0.07 |
| 18:1 | 0.19 | 0.07 | 0.01 |
| 18:2 | 0.34 | 0.52 | 0.09 |
| 20:4 | 0.04 | 0.27 | 0.32 |
| 22:6 | 0.17 | 0.01 | 0.51 |

A fit to the proportional incorporation model is performed on 10000 random datasets. An empirical p-value is assigned as. We get p-value = 0.028, which is below the threshold 0.05, suggesting that the model performance is not likely random.
